# The Association Between Early Drinking and Dependence Varies by Drinking Context

**DOI:** 10.3389/fnbeh.2020.00017

**Published:** 2020-03-05

**Authors:** Karina Conde, Raquel I. Peltzer, Paula V. Gimenez, Mariana Cremonte

**Affiliations:** Institute of Basic, Applied Psychology and Technology (IPSIBAT), National Scientific and Technical Research Council (CONICET), National University of Mar del Plata (UNMdP), Mar del Plata, Argentina

**Keywords:** alcohol, early drinking, dependence, alcohol policy, males, females

## Abstract

Evidence regarding the association between early drinking (ED) and later dependence is controversial. It has been alternately hypothesized that ED either plays a causal role in the development of dependence or that it is an early marker of increased psychosocial vulnerabilities. Despite a clear rationale for delaying youth consumption, it is important to discern this relationship. However, most epidemiological evidence comes from individual studies and high-income countries. If there is a causal link between ED and dependence, an association at the aggregate level would be expected. Furthermore, if the link is due to biological mechanisms, the association should be rather invariable regardless of the drinking context, while if the association is due to psychosocial factors, a wider variability is to be expected. We explored whether the association between ED and dependence varied across countries clustered by their shared contextual drinking characteristics. We used data from 169 countries from the Global Information System on Alcohol and Health of the World Health Organization: ED, alcohol dependence, heavy episodic drinking (HED), actual drinkers, and alcohol policy. To cluster countries by their shared drinking characteristics (prevalences of HED and actual drinkers, and alcohol policy), we used, sequentially, two multivariate data reduction techniques: a multiple correspondence analysis (MCA) and a hierarchic classification. To estimate the association between ED and alcohol dependence, beta regressions were performed, and then adjusted by country income-level and repeated by gender. The results indicated four country clusters: primarily abstainers (class 1), low drinking countries (class 2), high drinking countries (class 3), and very high drinking countries (class 4). Positive relationships between ED and alcohol dependence were found for all the countries in the world and for those in classes 1 and 2. No significant relationships were found for class 3 or class 4. These results were similar for males, but not for females, where no significant relationships were found after adjusting for income level. The association between ED and dependence varies according to the drinking context. Our findings either suggest that the ED–dependence association may be due to individual or environmental vulnerabilities that promote consumption outside cultural norms or that, if there is a causal link between ED and dependence, it is strongly moderated by psychosocial characteristics.

## Introduction

Evidence regarding the association of early drinking (ED) and the development of later problems is highly controversial (Frøydis et al., 2019). While many cross-sectional studies find an association between ED and later problems (e.g., dependence), longitudinal evidence accounting for confounders yields contradictory evidence (Connor et al., 2019). While there is a clear rationale for delaying consumption among youth, it is equally important to discern the nature of the relationship for theoretical and policy reasons. It has been alternately hypothesized that ED either plays a causal role in the development of later dependence or that it is simply an early marker of increased genetic and psychosocial vulnerabilities (Connor et al., 2019). Some authors have stated that, if there was a causal link between ED and dependence, an association at the aggregate level would be expected as self-selection and other biases present in individual-level studies would be absent. For this reason, some authors (Norström and Skog, 2001; Rossow, 2006) had pointed to the value of aggregate data for judging the plausibility of individual-level relationships when selection effects might be at play. However, as far as we know, available data from various countries, among them those from the ESPAD Study, failed to find such an association (Hibell et al., 2004; Kuntsche et al., 2016). Furthermore, if a causal relationship is to be assumed, and it is due to biological mechanisms (e.g., neurotoxic effects of ED), instead of social ones (e.g., through changing the social role of those drinking early; Frøydis et al., 2019), one would expect the association between ED and dependence to be rather invariable across countries; conversely, if the association is due to psychosocial factors, a wider variability among countries with different drinking contexts and policies is to be expected.

Most evidence about the association between ED initiation and the development of dependence comes from high-income countries, despite other world’s regions depicting most of the alcohol-related harms. This gap in the evidence deepens the inequality between regions. Furthermore, the lack of studies from low- and middle-income countries has been indicated as a limitation of the available evidence, as the consequences of ED could vary cross-culturally (Maimaris and McCambridge, 2014; Frøydis et al., 2019).

Additionally, although alcohol use among females has increased over time and ED has grown among them, evidence regarding the link between ED and later trajectories of alcohol use and related problems between genders has also been contradictory (Tomek et al., 2016).

We aimed to explore whether the association between ED and dependence varies across countries clustered by their shared contextual drinking characteristics and national alcohol policies. If there is a causal link between ED and dependence, we expect to find an association at the aggregate level. Furthermore, if the causal mechanism is of a biological nature, we expect this association to be fairly even among clusters of countries unrestrictedly of their drinking context. Conversely, if the relationship is mainly due to psychosocial factors, we expect the strength of the association between ED and dependence to vary. Specifically, we would estimate a stronger link between ED and dependence among those countries with drier cultures as ED in those countries would be a stronger marker of psychosocial vulnerabilities that lead youth to drink beyond what is normative in that context; contrarily, we would expect a weaker association among the most permissive countries where ED is not outside the limits of acceptable drinking behavior. Given that countries’ socioeconomic level could confound these associations (varying levels of early dependence detection or access to treatment, for instance), we will adjust by countries’ socioeconomic level. Lastly, since the ED–dependence association might differ between genders (Tomek et al., 2016), we will characterize the association by gender.

## Materials and Methods

### Design and Procedure

For this cross-sectional study, we used the global data available at the Global Information System on Alcohol and Health (GISAH) of the World Health Organization (2018a)^1^. Information of the GISAH measures comes from different sources, such as governmental reports, public statistics, local projects, and surveys and comprises more than 225 states (Poznyak et al., 2014). Here, data were available from 169 countries and missing from 25, which were, thus, excluded from the analyses. The gathered information included the following:

*Early Drinking (ED)*: The percentage of those who were current alcohol drinkers between the ages of 15 and 19 years during the past 12 months, in each country.

*Alcohol Dependence*: The percentage of those (15 years or older) with a diagnosis of alcohol dependence according to the International Classification of Diseases (ICD) during the past 12 months, in each country.

*Heavy Episodic Drinking (HED)*: The percentage of adults (15 years or older) who had at least 60 g of pure alcohol (approx. six standard alcoholic drinks) on at least one occasion in the last 30 days, in each country.

*Actual Drinkers*: The percentage of those who consumed any alcohol in the past 12 months, for each country.

*Alcohol Policy*: Whether the country adopted a written alcohol policy for reducing the burden on alcohol (yes/no/only at the subnational level/consumption prohibited).

We also used the following socioeconomic data from the World Bank (2019)^2^.

*Income Level*: Classification of the World Bank (i.e., low, middle-low, upper-middle, and high) based on the national income per person, for each country.

*Gini Index*: A measure of how much the distribution of income deviates from perfect inequality (100) and absolute equality (0), in each country.

### Data Analyses

To cluster countries by their shared drinking characteristics, we used, sequentially, two multivariate data reduction techniques: a multiple correspondence analysis (MCA) and a hierarchic classification. MCA is a valuable tool for the description and visualization of relationships in complex categorical data without the need of distributional assumptions (Greenacre, 2017; Quail et al., 2017). For factor analysis, the active variables are those that contribute to the variance (inertia) in the data set and to the formation of factorial axes. We considered as active variables the following: actual drinking prevalence, heavy episodic drinking (HED) prevalence, and whether there is a written alcohol policy in place. Since written policy was a nominal variable, the first two variables (drinking and HED prevalences) were divided into quartiles; hence, 1 was the lowest prevalence and 4 the highest. Based on the factor analysis, the classification was carried out on the three main axes, and a partition was subsequentially performed.

To estimate the association between ED and alcohol dependence, we performed first descriptive analyses, and then, beta regressions (using logit link and maximum likelihood estimator) for all the countries together and also within each class. Beta regressions are a suitable approach for beta-distributed data, such as percentages, where a linear regression model is not accurate. In order to improve interpretation, we exponentiated the log odds of significant relationships to obtain the odds ratios. For regressions, the percentage of alcohol dependence was the outcome variable, and the percentage of ED was set as the predictor. The analyses were performed adjusting by income level and the Gini index, but because of multicollinearity among them, and the results being no different with either measure (not shown), only income level was used. These analyses were repeated by gender (i.e., percentage of alcohol dependence and early alcohol consumption in males and females). For each regression, we used diagnostic plots to assess the regression assumptions, i.e., no detectable patterns among Pearson’s and deviance residuals (not shown). All regressions satisfied such conditions. Pseudo *R*^2^ was obtained to estimate the goodness of fit.

For data management and analyses, the Système Portable pour l’Analyse de Données Numériques (SPAD-N) version 4.1 for Windows (Lebart et al., 1983) and the R software version 3.5.3 for Windows, package betareg (Zeileis et al., 2016), were used.

## Results

### Clustering of Countries by Their Drinking Characteristics

**Figure 1:**
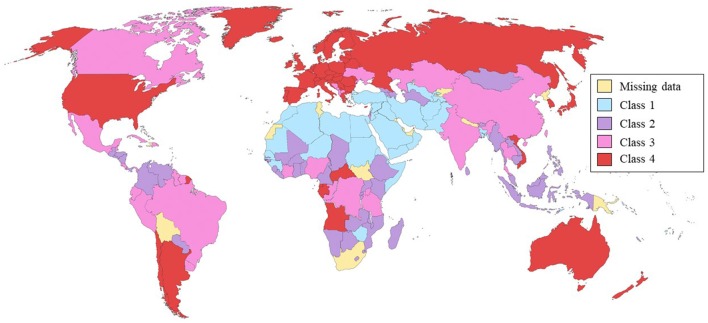
Countries’ clusters by actual drinking prevalence, heavy episodic drinking (HED) prevalence, and national alcohol policy.

Countries were clustered by their drinking characteristics (prevalence of HED and of actual drinkers, and alcohol policy) through factor analyses. The first three factorial axes were retained, accounting for 63.32% of the total variance. Hierarchic classification and partition resulted in four distinctive classes of drinking contexts and national policies. Figure 1 shows the countries’ pertinence to each cluster.

The first class (1/4; *n* = 33, 19.5%) clustered those countries with the lowest percentages of HED. Almost all of the countries in this class (96.9%) had very low percentages of actual drinkers. All the countries where consumption is prohibited were clustered here. This class seems to comprise those countries where abstention is the norm and could be considered *primarily abstainers*. The second class (2/4; *n* = 50, 29.6%) included those countries where HED was low; 97.6% of those with low HED prevalences and 97.5% of those with low drinking prevalences were clustered here. More than half of the countries in this class (62%) did not have a written alcohol policy. Countries in this class could be regarded as *low drinking* countries. The third class (3/4; *n* = 38, 22.5%) comprised those countries with high prevalences of HED (92% of those with HED on the third quartile were classified here) and of drinkers (89.5% of countries with a prevalence of drinkers on the third quartile were clustered here). More than half (66%) of the countries that had alcohol policies only at the subnational level were clustered in this class. These countries could be regarded as *high drinking* countries. The fourth class (4/4; *n* = 48, 28.4%) included those countries where the prevalence of HED was very high (91% of those countries were clustered here) and the prevalence of drinkers was also very high (89% of those countries were classified in this cluster). Most of the countries in this class (81%) had written alcohol policies. The countries in this class could be considered *very high drinking* countries (Supplementary Table S1).

### Description of ED, Dependence, and Income Level in Each Country Cluster

An increase in ED was observed in classes 1–4, ranging from 5% in class 1 to almost 60% in class 4. An increase in alcohol dependence was also found, but not as marked as that of ED. Regarding income level, countries in classes 1 and 2 were mostly low or middle-low income, countries in class 3 were mostly middle-low or upper-middle, while class 4 was mainly composed of high-income-level countries. These results are presented in Table 1.

**Table 1:** Early drinking (ED), alcohol dependence, and income level in each country class (by actual drinking prevalence, heavy episodic drinking prevalence, and national alcohol policy).

	*n*	Percentage	M (SD)
*Class 1 (n = 33)*			
ED			4.75 (4.73)
Alcohol dependence			0.86 (1.01)
Income level		
Low	13	39	
Middle-low	13	39	
Upper-middle	4	12	
High	3	9	
*Class 2 (n = 50)*			
ED			18.20 (4.25)
Alcohol dependence			2.04 (0.90)
Income level		
Low	22	44	
Middle-low	20	40	
Upper-middle	8	16	
High	−	−	
*Class 3 (n = 38)*			
ED			34.89 (7.97)
Alcohol dependence			2.69 (0.89)
Income level		
Low	3	8	
Middle-low	10	26	
Upper-middle	18	47	
High	7	18
*Class 4 (n = 48)*			
ED			57.68 (11.45)
Alcohol dependence			3.7 (2.5)
Income level		
Low	2	4
Middle-low	2	4
Upper-middle	11	23
High	33	69

### Relationships Between ED and Dependence for All Countries and by Country Cluster and Gender

The regression coefficients between ED and alcohol dependence for all countries and within each country cluster, unadjusted and adjusted by income level, are shown in Table 2 and, for each gender, in Table 3. A positive relationship was found between ED and alcohol dependence for all the countries (OR = 5.58, adjusted by income OR = 7.46), class 1 (OR = 323.76, adjusted by income OR = 419.89), and class 2 (OR = 98.49, adjusted by income OR = 29.08). No significant relationships were found between ED and alcohol dependence in class 3 or in class 4. These results were similar for males, but not for females. For females in all the countries combined, a positive association was found (OR = 8.01, adjusted by income OR = 5.64), but no significant relationship was found in class 1; in class 2, a relationship was found only in unadjusted regressions (OR = 5767.53). For males in all countries combined, a positive association was found (OR = 4.35, adjusted by income OR = 6.29), in class 1 (OR = 78.26, adjusted by income OR = 88.23), and class 2 (OR = 16.61, adjusted by income OR = 8.25).

**Table 2:** Beta regression analyses for the relationship between alcohol dependence and early drinking (ED) for all countries and by each country class.

	Estimate (95% CI)	*φ*	*R*^2^
*Total* (*n* = 194)			
ED	1.72 (1.32–2.11)***	93.53***	0.29
ED^1^	2.01 (1.43–2.58)***	94.46***	0.31
*Class 1* (*n* = 33)			
ED	5.78 (0.59–10.98)*	149.49***	0.22
ED^1^	6.04 (0.39–11.69)*	149.83***	0.23
*Class 2* (*n* = 50)			
ED	4.59 (1.69–7.5)**	234.68***	0.15
ED^1^	3.37 (0.42–6.32)*	261.24***	0.24
*Class 3* (*n* = 38)			
ED	0.07 (−1.74–1.88)	170.4***	0.01
ED^1^	−0.83 (−2.75–1.08)	200.32***	0.11
*Class 4* (*n* = 48)			
ED	0.88 (−0.68–2.43)	61.52***	0.03
ED^1^	1.67 (−0.36–3.71)	63.14***	0.05

The outcome variable was alcohol dependence in all the regressions. φ: Precision parameter. *p < 0.05, **p < 0.01, ***p < 0.001. ^1^Adjusted by country income level.

**Table 3:** Beta regression analyses for the relationship between alcohol dependence and early drinking (ED) for all countries and each country class by gender.

	Female			Male		
	Estimate (95% CI)	*φ*	*R*^2^	Estimate (95% CI)	*φ*	*R*^2^
*Total* (*n* = 194)						
ED	2.08 (1.61–2.55)***	186.03***	0.29	1.47 (1.11–1.83)***	55.37***	0.29
ED^1^	1.73 (1.07–2.39)***	186***	0.31	1.84 (1.33–2.35)***	57.03***	0.33
*Class 1* (*n* = 33)						
ED	4.21 (−4.54–12.95)	605.3***	0.05	4.36 (0.67–8.05)*	77.37***	0.24
ED^1^	4.74 (−4.67–14.14)	607.4***	0.05	4.48 (0.39–11.69)*	77.43***	0.24
*Class 2* (*n* = 50)						
ED	8.66 (1.99–15.34)*	231.7***	0.11	2.81 (0.83–4.78)**	167.53***	0.13
ED^1^	5.59 (−1.05–12.22)	284.8***	0.27	2.1 (0.06–4.13)*	179.37***	0.19
*Class 3* (*n* = 38)						
ED	0.72 (−1.77–3.21)	194.6***	0.01	−0.3 (−1.88–1.27)	119.95***	0.01
ED^1^	−1.01 (−3.42–1.41)	286.59***	0.28	−0.91 (−2.65–0.82)	131.19***	0.07
*Class 4* (*n* = 48)						
ED	1.77 (0.33–3.20)	149.83***	0.18	0.33 (−1.29–1.96)	33.16***	0.01
ED^1^	1.55 (−0.19–3.31)	150.48***	0.18	1.44 (−0.87–3.75)	34.11***	0.03

The outcome variable was alcohol dependence. φ: Precision parameter. *p < 0.05, **p < 0.01, ***p < 0.001. ^1^Adjusted by country income level.

## Discussion

In this article, we aimed to explore, first, whether there is an association between ED and dependence at the aggregate level as aggregate level data would not be as affected by selection bias as individual-level studies. Overall, this is for the world population, and for males and females separately, we found a moderate/strong association between ED and dependence. This finding is not surprising and concurs with other cross-sectional individual-level data. However, this evidence is not without divergence and so far has come almost exclusively from high-income countries (Maimaris and McCambridge, 2014; Kuntsche et al., 2016).

### Clustering of Countries by Their Drinking Characteristics

In order to determine whether the association varied according to the drinking context, which we would expect if the association is due to psychosocial factors rather than biological, countries were clustered by their drinking characteristics. For that purpose, we applied, sequentially, two data reduction techniques that clustered countries by their percentage of actual drinkers, percentage of HED during the last month, and the national alcohol policy. We found four distinctive clusters of countries: two of them where abstention or infrequent drinking seems to be the norm and two where drinking is frequent. Notably, although socioeconomic data were not used for clustering, the first two classes were mostly formed by low- and middle-low-income countries, while the third was mainly by upper-middle-income countries and the fourth (depicting the highest level of drinking and HED) mostly by high-income countries. As expected, we found increasingly higher prevalences of ED and dependence in each of the four classes.

### Relationships Between ED and Dependence for All Countries and by Country Cluster and Gender

Second, we explored whether the relationship between ED and dependence varied across countries clustered by their shared drinking characteristics. We expected that, if the causal mechanism was of a biological nature, the association between ED and dependence would be fairly even among clusters of countries. Conversely, if the relationship was mainly due to psychosocial factors, we expected the strength of the association between ED and dependence to vary. Specifically, we estimated a stronger link between ED and dependence among those countries with drier cultures (by our results, primarily abstainers and low drinking classes) as ED in those countries would be a stronger marker of psychosocial vulnerabilities that lead youth to drinking beyond what is normative in that context; contrarily, we would expect a weaker association among the most permissive countries where ED is not outside the limits of acceptable drinking behavior (namely, high drinking and very high drinking classes). Our results confirmed this last hypothesis. We found positive and strong relationships between ED and alcohol dependence for the two clusters of countries where abstention or low infrequent drinking seems to be normative, while no significant relationships were found in those classes where drinking seems to be the common practice (the high drinking and very high drinking classes). Furthermore, the association was somewhat stronger for the primarily abstainers class (with higher abstention rates) than for the low drinking class. These results indicate that the association between ED and dependence varies according to the cultural context. This finding is supported by other authors (Maimaris and McCambridge, 2014; Kuntsche et al., 2016; Frøydis et al., 2019) who question the assumption that the association between ED and later problems is independent of cultural norms and national alcohol policies. Given that a solid association is a requirement for causation, our results would not lend empirical basis to a strong causal link between ED and dependence. Our findings suggest, at least at the epidemiological level, and as found in many of the few prospective studies that control for third variables (Aiken et al., 2018), that the association may be due to genetic, biologic, or psychosocial vulnerabilities that promote consumption outside cultural norms. However, since there is evidence of biological mechanisms that increase the risk of developing alcohol dependence after ED (Buchmann et al., 2009; Varlinskaya et al., 2020) and that ED may exacerbate genetic influences (Agrawal et al., 2009), an alternative possibility is that the causal link is anteceded by genetic psychosocial characteristics and also strongly moderated by them. That is, those with certain vulnerabilities (genetics for instance) may deviate from cultural norms and initiate early into consumption (marker hypothesis). ED may, in turn, put into place biological mechanisms which increase their risk of pathological consumption (causal link hypothesis), and depending on psychosocial and environmental characteristics, they may, or not, progress into dependence. Therefore, future research must imply large longitudinal studies that include different cultures and drinking contexts in order to assess the correspondence of changes in ED and alcohol-related problems over the years.

Given that countries’ socioeconomic level could confound these associations (varying levels of ED detection or access to treatment, for instance), we adjusted by each country socioeconomic level. When adjusted by income, surprisingly, the association was stronger for the whole sample and for the primarily abstainers class, while it was attenuated for the low drinking class, suggesting that the country’s socioeconomic level may be a moderator variable, by, for instance, facilitating or inhibiting the progression from drinking to dependence. Future studies should explore how societal socioeconomic conditions could affect drinking trajectories.

Lastly, since the ED–dependence association might differ between genders (Tomek et al., 2016), we characterized the association by gender. The results were similar for males, but not for females. For females in all the countries combined, a positive association was found (also attenuated when controlling for socioeconomic level), which indicated that, for each percentile increase in ED, there was almost a fivefold increase in the prevalence of dependence. However, we found no association between ED and dependence among females for any of the country clusters. Although consumption among women has increased over time, bearing in mind that we only found an association between ED and dependence among those country clusters where drinking is very infrequent, one possible rationale for this finding is that data for females in those countries were insufficient and that the analysis lacked statistical power. However, these findings suggest that, even if there was an association we could not detect with the available data, that association was weak. Conversely, the results for males were similar to those for the total sample, not surprisingly given that both consumption and dependence are higher among men (World Health Organization, 2018b), and thus, it is men’s consumption which accounts for most of the whole sample variability.

The results presented here are not without limitations. The data we analyzed as ED were the prevalences of drinking on those aged 15–19 years in each country. First of all, these being aggregated cross-sectional data, we should bear in mind that those with ED are not the same individuals as those with alcohol dependence. Nonetheless, aggregated data have the advantage of not being affected by self-selection bias, a common limitation of longitudinal studies. Furthermore, some evidence signals that different ED measures (such as first sip, first drunkenness, or initiation of regular drinking) might yield different results (Morean et al., 2018). It has also been suggested (Maggs et al., 2019) that there might be differences in whether drinking initiation occurs during early adolescence or during childhood (i.e., before 11 years of age), and we did not consider those factors. However, research indicates (Connor et al., 2019) that the onset of regular drinking would be a more solid measure of later problems than others (such as age of first sip), giving support to the measure we used here.

To conclude, and despite limitations, we provide evidence that the ED–dependence association is not independent of cultural drinking norms and national alcohol policies. Furthermore, the results expose aspects that may add to the understanding of the relationship between ED and later problems. Such characteristics relate both to the individual and to the context in which alcohol is consumed, whether they are of a cultural nature such as gender, psychosocial such as social norms, or macroeconomic such as a country’s income level.

## Data Availability Statement

Publicly available datasets were analyzed for this study. The datasets can be found here: World Bank Open Data, https://data.worldbank.org/ and here: Global Information System on Alcohol and Health (GISAH), https://apps.who.int/gho/data/node.main.GISAH?lang=en.

## Author Contributions

KC, RP, and MC contributed to the conception and design of the study. KC organized the database. KC and RP performed the statistical analysis. All authors wrote sections of the manuscript. All authors contributed to the manuscript revision and read and approved the submitted version.

## Conflict of Interest

The authors declare that the research was conducted in the absence of any commercial or financial relationships that could be construed as a potential conflict of interest.

## Footnotes

^1^https://apps.who.int/gho/data/node.main.GISAH?lang=en

^2^https://data.worldbank.org/

## References

[B1] Agrawal A. Sartor C. E. Lynskey M. T. Grant J. D. Pergadia M. L. Grucza R.. (2009). Evidence for an interaction between age at first drink and genetic influences on DSM-IV alcohol dependence symptoms. Alcohol. Clin. Exp. Res. 33, 2047–2056. 10.1111/j.1530-0277.2009.01044.x 19764935 PMC2883563

[B2] Aiken A. Philip J. C. Wadolowski M. Hutchinson D. Najman J. M. Slade T.. (2018). Age of alcohol initiation and progression to binge drinking in adolescence: a prospective cohort study. Alcohol. Clin. Exp. Res. 42, 100–110. 10.1111/acer.13525 29160941

[B3] Buchmann A. F. Schmid B. Blomeyer D. Becker K. Treutlein J. Zimmermann U. S.. (2009). Impact of age at first drink on vulnerability to alcohol-related problems: testing the marker hypothesis in a prospective study of young adults. J. Psychiatr. Res. 43, 1205–1212. 10.1016/j.jpsychires.2009.02.006 19332346

[B4] Connor J. P. Weier M. Hall W. D. (2019). “The age of onset of alcohol use disorders,” in Age of Onset of Mental Disorders, eds Girolamo G. D. McGorry P. D. Sartorius N. (Switzerland: Springer), 169–182.

[B5] Frøydis E. Evans-Whipp T. Kjeldsen A. Toumbourou J. W. von Soest T. (2019). Predicting hazardous drinking in late adolescence/young adulthood from early and excessive adolescent drinking-a longitudinal cross-national study of norwegian and australian adolescents. BMC Public Health 19:790. 10.1186/s12889-019-7099-0 31226962 PMC6588913

[B6] Greenacre M. (2017). Correspondence Analysis in Practice. Boca Raton, FL: Chapman and Hall/CRC.

[B7] Hibell B. Andersson B. Bjarnason T. Ahlström S. Balakireva O. Kokkevi A. (2004). The ESPAD Report 2003: Alcohool and Other Drug use Among Students in 35 European Countries. Stockholm: Swedish Council for Information on Alcohol and Other Drugs.

[B8] Kuntsche E. Rossow I. Engels R. Kuntsche S. (2016). Is ‘age at first drink’a useful concept in alcohol research and prevention? we doubt that. Addiction 111, 957–965. 10.1111/add.12980 26147610

[B9] Lebart L. Morineau A. Bécue M. Haeusler L. (1983). SPAD. T, Système Portable Pour l’Analyse Des Données Textuelles. Manuel De l’utilisateur. Paris: CISIA.

[B10] Maggs J. L. Staff J. Patrick M. E. Wray-Lake L. (2019). Very early drinking: event history models predicting alcohol use initiation from age 4 to 11 years. Addict. Behav. 89, 121–127. 10.1016/j.addbeh.2018.09.030 30290300

[B11] Maimaris W. McCambridge J. (2014). Age of first drinking and adult alcohol problems: systematic review of prospective cohort studies. J. Epidemiol. Community Health 68, 268–274. 10.1136/jech-2013-203402 24249000 PMC4158030

[B12] Morean M. E. L’Insalata A. Butler E. R. McKee A. Krishnan-Sarin S. (2018). Age at drinking onset, age at first intoxication, and delay to first intoxication: assessing the concurrent validity of measures of drinking initiation with alcohol use and related problems. Addict. Behav. 79, 195–200. 10.1016/j.addbeh.2017.12.017 29304425 PMC5807182

[B13] Norström T. Skog O. J. (2001). Alcohol and mortality: methodological and analytical issues in aggregate analyses. Addiction 96, S5–S17. 10.1080/09652140020021143 11228078

[B14] Poznyak V. Fleischmann A. Rekve D. Rylett M. Rehm J. Gmel G. (2014). The world health organization’s global monitoring system on alcohol and health. Alcohol Res. 35, 244–249. 24881333 10.35946/arcr.v35.2.15 PMC3908716

[B15] Quail J. Osman M. Teare G. (2017). Multiple correspondence analysis is a useful tool to visualize complex categorical correlated data. Int. J. Popul. Data Sci. 1:099 10.23889/ijpds.v1i1.118

[B16] Rossow I. (2006). “Inferences of associations and implications for prevention: the case of early drinking onset,” in Understanding Choice, Explaining Behaviour: Essays in Honour of Ole-Jørgen Skog, eds Elster J. Gjelsvik O. Hylland A. Moene K. (Oslo, Norway: Oslo Academic Press), 259–272.

[B17] Tomek S. Bolland K. A. Bolland J. M. Hooper L. M. Church W. T. Bolland A. C. (2016). Age of alcohol initiation matters: examining gender differences in the recency and frequency of alcohol use across adolescence using a sample of impoverished minority adolescents. Youth Soc. 51, 120–145. 10.1177/0044118x16662749

[B18] Varlinskaya E. I. Hosová D. Towner T. Werner D. F. Spear L. P. (2020). Effects of chronic intermittent ethanol exposure during early and late adolescence on anxiety-like behaviors and behavioral flexibility in adulthood. Behav. Brain Res. 378:112292. 10.1016/j.bbr.2019.112292 31626849 PMC7261490

[B19] World Bank (2019). World Bank Open Data. Available online at: https://data.worldbank.org/. Accessed July 16, 2019.

[B21] World Health Organization (2018a). Global Information System on Alcohol and Health (GISAH). World Health Organization Available online at: https://apps.who.int/gho/data/node.main.GISAH?lang=en. Accessed April 27, 2018.

[B20] World Health Organization (2018b). Global Status Report on Alcohol and Health 2018. Geneva: World Health Organization.

[B22] Zeileis A. Cribari-Neto F. Gruen B. Kosmidis I. Simas A. B. Rocha A. V. (2016). Package ‘Betareg’. R Package Available online at: https://cran.r-project.org/web/packages/betareg/betareg.pdf.

