# Supplementary material for: The Association Between Early Drinking and Dependence Varies by Drinking Context
Source: Front Behav Neurosci. 2020 Mar 5;14:17. doi: 10.3389/fnbeh.2020.00017 (PMC7066110; doi:10.3389/fnbeh.2020.00017)
Supplement: Supplementary file 1 [file Table_1.pdf]

Supplementary Material

Table S1. Clustering of countries by their drinking characteristics and alcohol policies.

				Characteristics								
				Heavy episodic drinking			Drinkers			Alcohol policy		
Class	Denomination	n	%	Values	%[1]	%[2]	Values	%[1]	%[2]	Values	%[1]	%[2]
1/4	Abstainers	33	19.5	Lowest percentages of HED	100	89	Lowest percentages of actual drinkers	96.9	86.4	Consumption prohibited	24	100
2/4	Low drinking countries	50	29.6	Low percentage of HED	78	97.5	Low drinking percentage	80	97.5	None	62	41.9
3/4	High drinking countries	38	22.5	High percentage of HED	92	77.7	High drinking percentage	89	77	Subnational level	15.8	66.6
4/4	Very high drinking countries	48	28,4	Very high percentage of HED	91.6	93.6	Very high drinking percentage	89.5	91.4	Yes	81.2	50

% [1]= % of those presenting the characteristic within the class.
%[2]= % of the class presenting that characteristic within those with that value.
Only included $p < 0.005$ and V-Test > 2.65
